# Lymphocytic Choriomeningitis Virus Lineage V in Wood Mice, Germany

**DOI:** 10.3201/eid3002.230868

**Published:** 2024-02

**Authors:** Calvin Mehl, Olayide Abraham Adeyemi, Claudia Wylezich, Dirk Höper, Martin Beer, Cornelia Triebenbacher, Gerald Heckel, Rainer G. Ulrich

**Affiliations:** Friedrich-Loeffler-Institut, Greifswald-Insel Riems, Germany (C. Mehl, O.A. Adeyemi, C. Wylezich, D. Höper, M. Beer, R.G. Ulrich);; German Center for Infection Research, Partner Site Hamburg-Lübeck-Borstel-Riems, Germany (C. Mehl, R.G. Ulrich);; Bavarian State Institute for Forest and Forestry, Freising, Germany (C. Triebenbacher);; University of Bern, Bern, Switzerland (G. Heckel)

**Keywords:** lymphocytic choriomeningitis virus, LCMV, lineage V, wood mice, *Apodemus sylvaticus*, meningitis/encephalitis, viruses, zoonoses, Germany

## Abstract

We identified a novel lineage of lymphocytic choriomeningitis virus, tentatively named lineage V, in wood mice (*Apodemus sylvaticus*) from Germany. Wood mouse–derived lymphocytic choriomeningitis virus can be found across a substantially greater range than previously thought. Increased surveillance is needed to determine its geographic range and zoonotic potential.

Lymphocytic choriomeningitis virus (LCMV; species *Mammarenavirus choriomeningitidis*) is a single-stranded RNA virus that has a bisegmented genome and ambisense coding strategy (*1*). LCMV is a zoonotic virus that causes encephalitis, meningitis, and sudden infant death syndrome in humans (*2*,*3*) and callitrichid hepatitis in New World primates (family Callitrichidae) (*4*). According to phylogenetic analysis, LCMV lineages I–IV are recognized. The most common, lineages I and II, are found worldwide (the house mouse, *Mus musculus*, is a reservoir host), whereas lineage III was found in 1 patient in Georgia, USA. Lineage IV was identified by sequencing small (S) RNA segments obtained from wood mice (*Apodemus sylvaticus*) found at 3 sites in southern Spain (*5*). That same study also reported the presence of LCMV antibodies in *M. musculus*, *M. spretus*, and *Rattus norvegicus* (Norway) rats in Spain (*5*). Similarly, LCMV-reactive antibodies have been found in wood mice from Austria (*6*) and in yellow-necked field mice (*Apodemus flavicollis*) and voles from Italy (*7*). LCMV reemerged in Germany in a captive golden lion tamarin (*Leontopithecus rosalia*) and sympatric wild *M. musculus domesticus* mice (*8*). We report the discovery of LCMV RNA in wood mice from Germany. 

High-throughput sequencing of pooled brain tissue from *Apodemus* spp. captured in southern Germany revealed the presence of LCMV sequence reads (Appendix). We tested brain tissue samples from each of those animals (4 yellow-necked field mice and 13 wood mice) separately by reverse transcription PCR (*9*). We found LCMV amplification products of the expected length only in 2 wood mouse samples (KS20/3119 and KS20/3122). In addition, we tested 132 rodents and shrews collected during 2005–2018, representing 5 species from the same geographic region in Bavaria, Germany, as the 2 LCMV RNA-positive animals. Those 132 animals were negative for LCMV RNA by using conventional panarenavirus reverse transcription PCR (Appendix Table 1, Figure 1). 

We captured all 134 animals (132 rodents and shrews plus 2 LCMV-positive wood mice) near natural forest or reforested areas at an altitude of 366–620 m by using line trapping. We placed traps 2 m apart within lines and 10 m between lines. We trapped animals 1 time per year for 2 consecutive nights during 2005–2018.

We assembled nearly complete sequences of LCMV large (L) and S RNA segments and host mitochondrial cytochrome b DNA from brain tissue of the 2 LCMV-positive wood mice and performed phylogenetic analyses. We deposited LCMV sequences obtained in this study in GenBank (accession nos. OR135709–12). The L (7,144 nt) and S (3,342 nt) sequences contained complete coding regions except for the first ≈55 nt and last ≈18 nt of the L segment and first ≈18 nt and last ≈24 nt of the S segment. For all 3 coding regions examined (L protein, glycoprotein, and nucleocapsid protein), virus sequences from the 2 mice formed a separate monophyletic clade (tentatively named lineage V) that is ancestral to all previously published LCMV sequences (Figure; Appendix Figures 2, 3) and highly divergent at the nucleotide and amino acid sequence levels (Appendix Table 2). Phylogenetic analysis of wood mouse mitochondrial cytochrome b sequences showed that both LCMV-positive animals clustered with *Apodemus sylvaticus* subclade 2b (Appendix Figure 4), the same subclade as the mice from Spain in which LCMV lineage IV was discovered (*5*).

**Figure:**
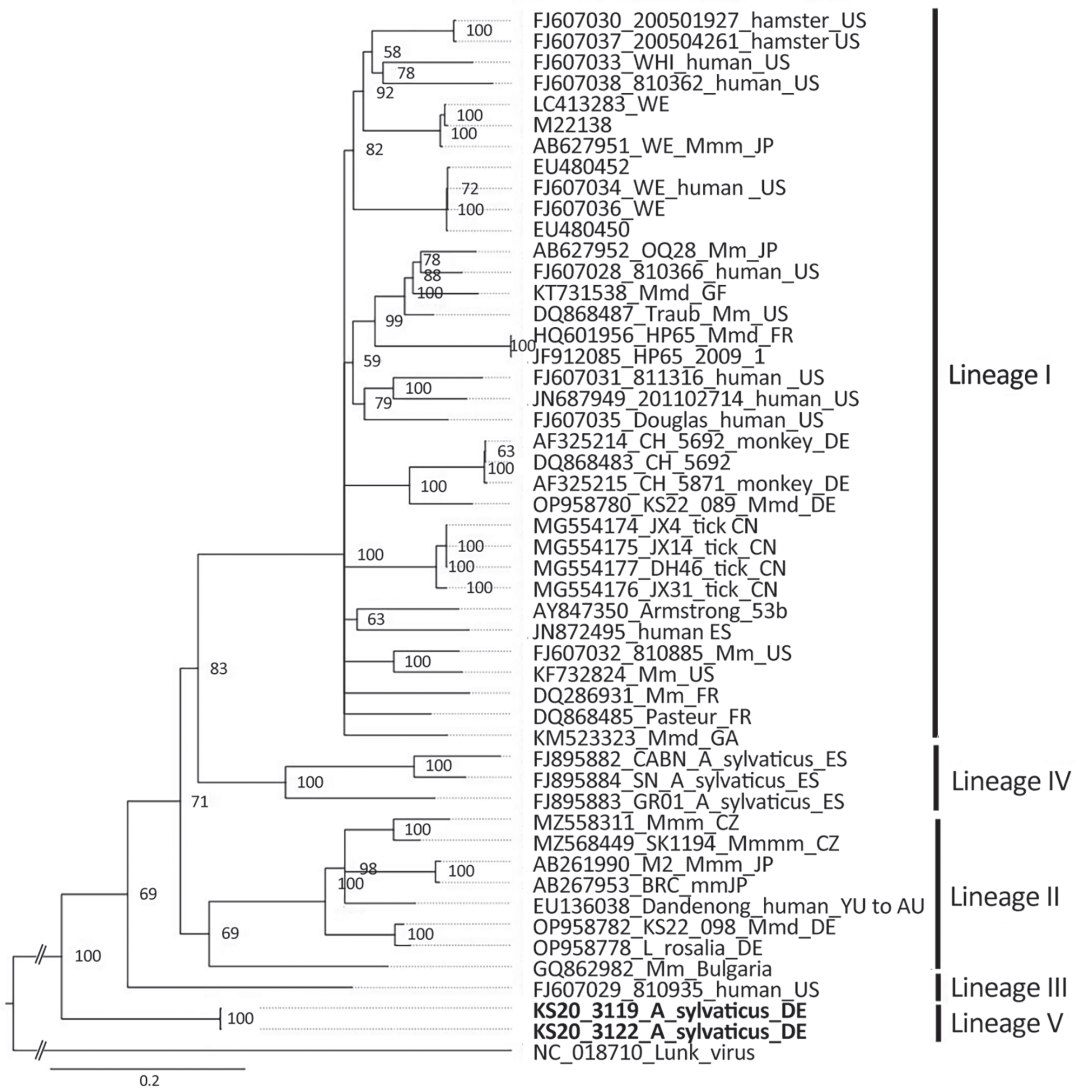
Phylogenetic analysis of the nucleocapsid protein encoding region of lymphocytic choriomeningitis virus lineage V identified in wood mice, Germany (boldface), and reference sequences. Bayesian inference method was used to analyze the 1,674-nt open reading frame corresponding to codons 1–558 without the stop codon. GenBank accession number, strain name, host species, and country of origin (if known) are shown. Roman numerals I–IV represent the different virus lineages as defined previously (*10*). Lunk virus from *Mus minutoides* mice was used as an outgroup. WE and Armstrong are laboratory strains of lymphocytic choriomeningitis virus. Scale bar indicates nucleotide substitutions per site. Asyl, *Apodemus sylvaticus*; AU, Australia; BG, Bulgaria; CN, China; CZ, Czech Republic; DE, Germany; ES, Spain; FR, France; GA, Gabon; GF, French Guiana; JP, Japan; Mm, *Mus musculus*; Mmm, *M. musculus musculus*; Mmd, *M. musculus domesticus*; SK, Slovakia; US, United States; YU, former Yugoslavia.

In conclusion, we identified a new LCMV lineage in wood mice from southern Germany. Unlike Dandenong virus, an unclassified mammarenavirus that falls within lineage II (both L and S segments), sequences from lineage V constitute their own distinct clade that is basal to other known LCMV lineages. Host mitochondrial DNA sequences indicated the wood mice from Germany belonged to the same clade as those in which LCMV lineage IV was previously identified in Spain. The serologic evidence of LCMV in wood mice from Italy (*7*) and Austria (*6*) combined with LCMV RNA detection in wood mice from Spain (*5*) and this study suggest that wood mouse-derived LCMV can be found across a substantially greater range than previously thought. Greater surveillance is needed to determine the geographic range and diversity of LCMV in small mammals and the potential infection risk to humans.

Appendix Additional information for lymphocytic choriomeningitis virus lineage V in wood mice, Germany.
