# Supplementary material for: Lymphocytic Choriomeningitis Virus Lineage V in Wood Mice, Germany
Source: Emerg Infect Dis. 2024 Feb;30(2):399–401. doi: 10.3201/eid3002.230868 (PMC10826776; doi:10.3201/eid3002.230868)
Supplement: Appendix — Additional information for lymphocytic choriomeningitis virus lineage V in wood mice, Germany. [file 23-0868-Techapp-s1.pdf]

Article DOI: https://doi.org/10.3201/eid3002.230868

EID cannot ensure accessibility for supplementary materials supplied by authors. Readers who have difficulty accessing supplementary content should contact the authors for assistance.

Lymphocytic Choriomeningitis Virus Lineage V in Wood Mice, Germany

Appendix

Additional Methods

Genetic screening of wood mice and other sympatric small mammals was performed by using a SuperScript III One-Step RT-PCR Kit (Thermo Fisher Scientific, https://www.thermofisher.com) and arenavirus primers (*1*). Brain tissues were homogenized and nucleic acids were isolated by using a NucleoMag VET kit (Macherey-Nagel, https://www.mn-net.com) and KingFisher Flex Purification System (Thermo Fisher Scientific). High-throughput sequencing was performed as previously described (*2*). Coding sequences of lymphocytic choriomeningitis virus large and small segments and host mitochondrial cytochrome b DNA were obtained through a de novo assembly with SPAdes version 3.13.0 (*3*). Sequences were aligned by using the ClustalW algorithm in BioEdit (*4*). The best-fit nucleotide substitution model was determined by using JModelTest2 (*5*,*6*). Thereafter, phylogenetic trees were constructed by using MrBayes v.3.2.7 (*7*). For all 3 coding regions, Bayesian tree reconstruction was performed by using a general time reversal substitution model with gamma distribution and a proportion of invariable sites. Twenty million generations were run; trees were sampled every 1000 generations, and the first 25% were discarded as burn-in. For maximum-likelihood tree construction of *Apodemus sylvaticus* cytochrome b, we used the general time reversal substitution model with gamma distribution and a proportion of invariable sites. Bootstraps were calculated from 1000 replications (*8*). LCMV sequences obtained in this study were deposited in GenBank and are available under the accession numbers OR135709–12.

References

1. Vieth S, Drosten C, Lenz O, Vincent M, Omilabu S, Hass M, et al. RT-PCR assay for detection of Lassa virus and related Old World arenaviruses targeting the L gene. Trans R Soc Trop Med Hyg. 2007;101:1253–64. PubMed https://doi.org/10.1016/j.trstmh.2005.03.018

2. Pfaff F, Breithaupt A, Rubbenstroth D, Nippert S, Baumbach C, Gerst S, et al. Revisiting rustrela virus: new cases of encephalitis and a solution to the capsid enigma. Microbiol Spectr. 2022;10:e0010322. PubMed https://doi.org/10.1128/spectrum.00103-22

3. Nurk S, Bankevich A, Antipov D, Gurevich AA, Korobeynikov A, Lapidus A, et al. Assembling single-cell genomes and mini-metagenomes from chimeric MDA products. J Comput Biol. 2013;20:714–37. PubMed https://doi.org/10.1089/cmb.2013.0084

4. Hall TA. BioEdit: a user-friendly biological sequence alignment editor and analysis program for Windows 95/98/NT. Nucleic Acids Symp Ser. 1999;41:95–98.

5. Guindon S, Gascuel O. A simple, fast, and accurate algorithm to estimate large phylogenies by maximum likelihood. Syst Biol. 2003;52:696–704. PubMed https://doi.org/10.1080/10635150390235520

6. Darriba D, Taboada GL, Doallo R, Posada D. jModelTest 2: more models, new heuristics and parallel computing. Nat Methods. 2012;9:772. PubMed https://doi.org/10.1038/nmeth.2109

7. Ronquist F, Teslenko M, van der Mark P, Ayres DL, Darling A, Höhna S, et al. MrBayes 3.2: efficient Bayesian phylogenetic inference and model choice across a large model space. Syst Biol. 2012;61:539–42. PubMed https://doi.org/10.1093/sysbio/sys029

8. Tamura K, Stecher G, Kumar S. MEGA11: molecular evolutionary genetics analysis version 11. Mol Biol Evol. 2021;38:3022–7. PubMed https://doi.org/10.1093/molbev/msab120

9. Albariño CG, Palacios G, Khristova ML, Erickson BR, Carroll SA, Comer JA, et al. High diversity and ancient common ancestry of lymphocytic choriomeningitis virus. Emerg Infect Dis. 2010;16:1093–100. PubMed https://doi.org/10.3201/eid1607.091902

10. Michaux JR, Libois R, Filippucci MG. So close and so different: comparative phylogeography of two small mammal species, the yellow-necked fieldmouse (*Apodemus flavicollis*) and the woodmouse (*Apodemus sylvaticus*) in the Western Palearctic region. Heredity. 2005;94:52–63. PubMed https://doi.org/10.1038/sj.hdy.6800561

Appendix Table 1. Number of animals tested for lymphocytic choriomeningitis virus*

Site	Species	Common name	No. positive/total no. tested
1	*Apodemus sylvaticus*	Wood mouse	0/2
2	*Apodemus sylvaticus*	Wood mouse	0/5
3	*Sorex araneus*	Common shrew	0/2
4	*Myodes glareolus* (syn. *Clethrionomys glareolus)*	Bank vole	0/3
5	*Apodemus sylvaticus*	Wood mouse	0/3
6	*Apodemus sylvaticus*	Wood mouse	2/3
	Myodes glareolus (syn. *Clethrionomys glareolus)*	Bank vole	0/29
	Microtus agrestis	Field vole	0/6
7	*Myodes glareolus* (syn. *Clethrionomys glareolus)*	Bank vole	0/3
8	*Sorex araneus*	Common shrew	0/1
9	*Microtus agrestis*	Field vole	0/9
	Sorex araneus	Common shrew	0/1
10	*Myodes glareolus* (syn. *Clethrionomys glareolus)*	Bank vole	0/18
	Sorex araneus	Common shrew	0/2
11	*Apodemus flavicollis*	Yellow-necked field mouse	0/28
	Myodes glareolus (syn. *Clethrionomys glareolus)*	Bank vole	0/9
	Microtus agrestis	Field vole	0/2
	Sorex araneus	Common shrew	0/2
12	*Apodemus sylvaticus*	Wood mouse	0/1
	Myodes glareolus (syn. *Clethrionomys glareolus)*	Bank vole	0/2
	Microtus agrestis	Field vole	0/3
Totals	*Apodemus sylvaticus*	Wood mouse	2/14
	Apodemus flavicollis	Yellow-necked field mouse	0/28
	Myodes glareolus	Bank vole	0/64
	Microtus agrestis	Field vole	0/20
	Sorex araneus	Common shrew	0/8

*Locations of sites are presented in Appendix Figure 1. syn, synonym.

Appendix Table 2. Nucleotide and amino acid sequence differences between the newly identified lineage V and recognized lineages of lymphocytic choriomeningitis virus*

	Large protein		Glycoprotein		Nucleoprotein	
Clade	Nucleotide	Amino acid	Nucleotide	Amino acid	Nucleotide	Amino acid
Lineage I	28.4 (29.2)	22.4 (24.1)	24.4 (25.5)	16.1 (18.5)	21.3 (23.5)	7.8 (11.8)
Lineage II	27.8 (28.4)	21.7 (22.2)	23.5 (24.0)	14.3 (15.7)	20.7 (21.4)	7.0 (7.9)
Lineage III	28.3 (28.3)	21.8 (21.8)	23.6 (23.6)	16.7 (16.7)	20.0 (20.0)	6.3 (6.3)
Lineage IV	ND	ND	25.3 (26.0)	17.7 (19.1)	20.4 (21.4)	8.9 (9.5)
Lunk virus outgroup	36.4 (36.4)	35.8 (35.8)	29.7 (29.7)	22.1 (22.1)	27.6 (27.6)	17.7 (17.7)

*Values are mean (maximum) % sequence differences. ND, no data available.

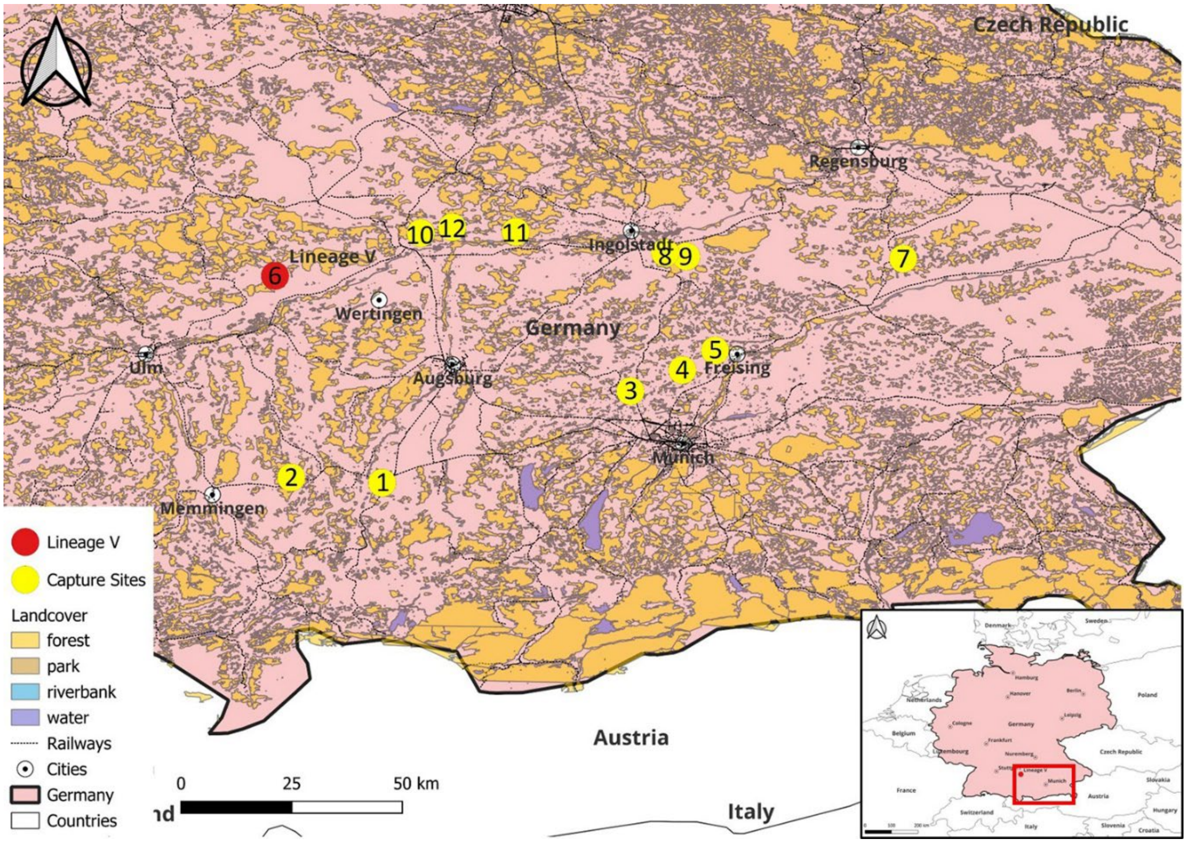

Appendix Figure 1. Map showing animal trapping site locations and land cover types in southern Germany. Red square in inset map indicates the area in southern Germany where trapping occurred. Yellow and red circles indicate capture sites in the main map. Red circle shows location where 2 *Apodemus sylvaticus* mice infected with lymphocytic choriomeningitis virus lineage V were found (site 6). Map was produced by using QGIS 3.32.0 (QGIS Geographic Information System, Open Source Geospatial Foundation Project, http://qgis.org). Data were obtained from OpenStreetMap (https://www.openstreetmap.org).

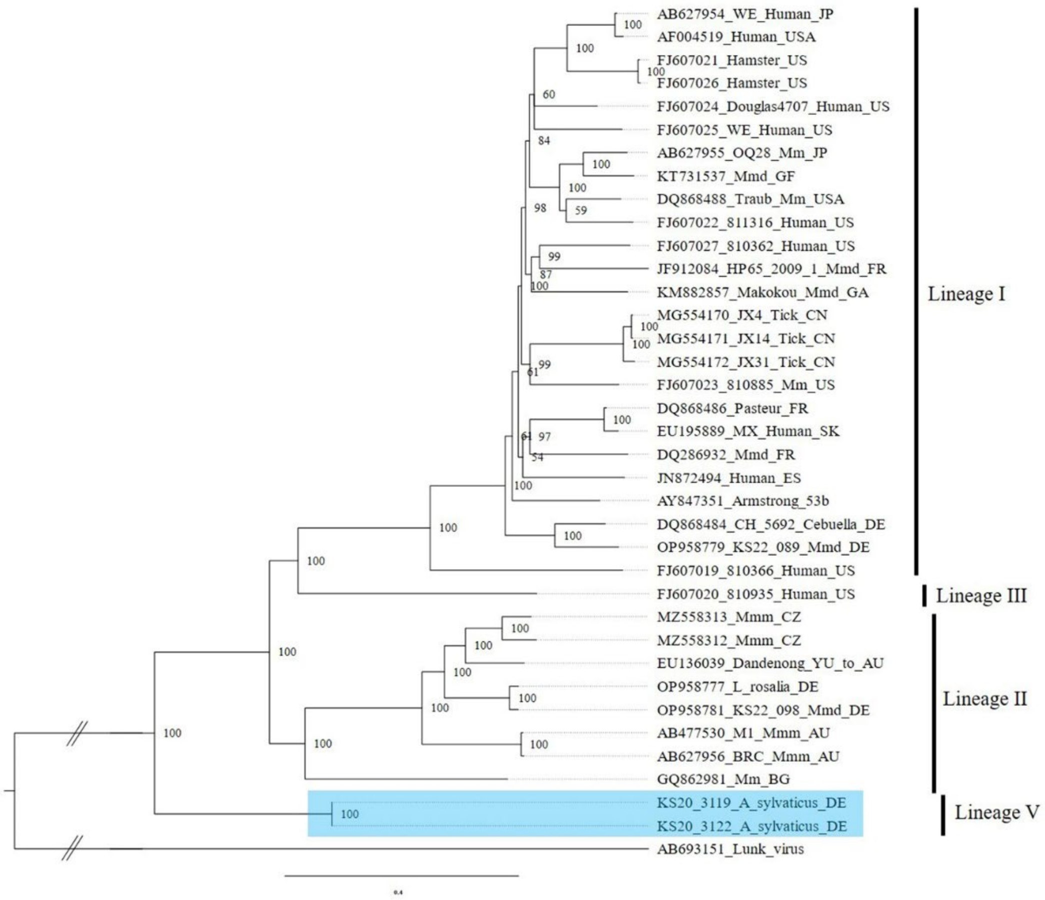

Appendix Figure 2. Phylogenetic analysis of the L protein encoding region of lymphocytic choriomeningitis virus lineage V in wood mice, Germany. Bayesian inference method was used to analyze 6,597 nt corresponding to codons 2–2210 without the start codon or the 10 codons (including the stop codon) at the 3′ end. Sequences from this study are highlighted in blue. Lunk virus from *Mus minutoides* mice was used as an outgroup. Sequence names are comprised of the GenBank accession number, strain name, host species, and country of origin (if known). Countries are represented by their international organization of standardization code. Roman numerals I–III represent the different virus lineages as defined previously (*9*). L-segment sequences for lineage IV were not available. WE and Armstrong are laboratory strains of lymphocytic choriomeningitis virus. Asyl, *Apodemus sylvaticus*; AU, Australia; BG, Bulgaria; CN, China; DE, Germany; ES, Spain; FR, France; GA, Gabon; GF, French Guiana; JP, Japan; Mm, *Mus musculus*; Mmm, *Mus musculus musculus*; Mmd, *Mus musculus domesticus*; SK, Slovakia; US, United States; YU, former Yugoslavia. Scale bar indicates nucleotide substitutions per site.

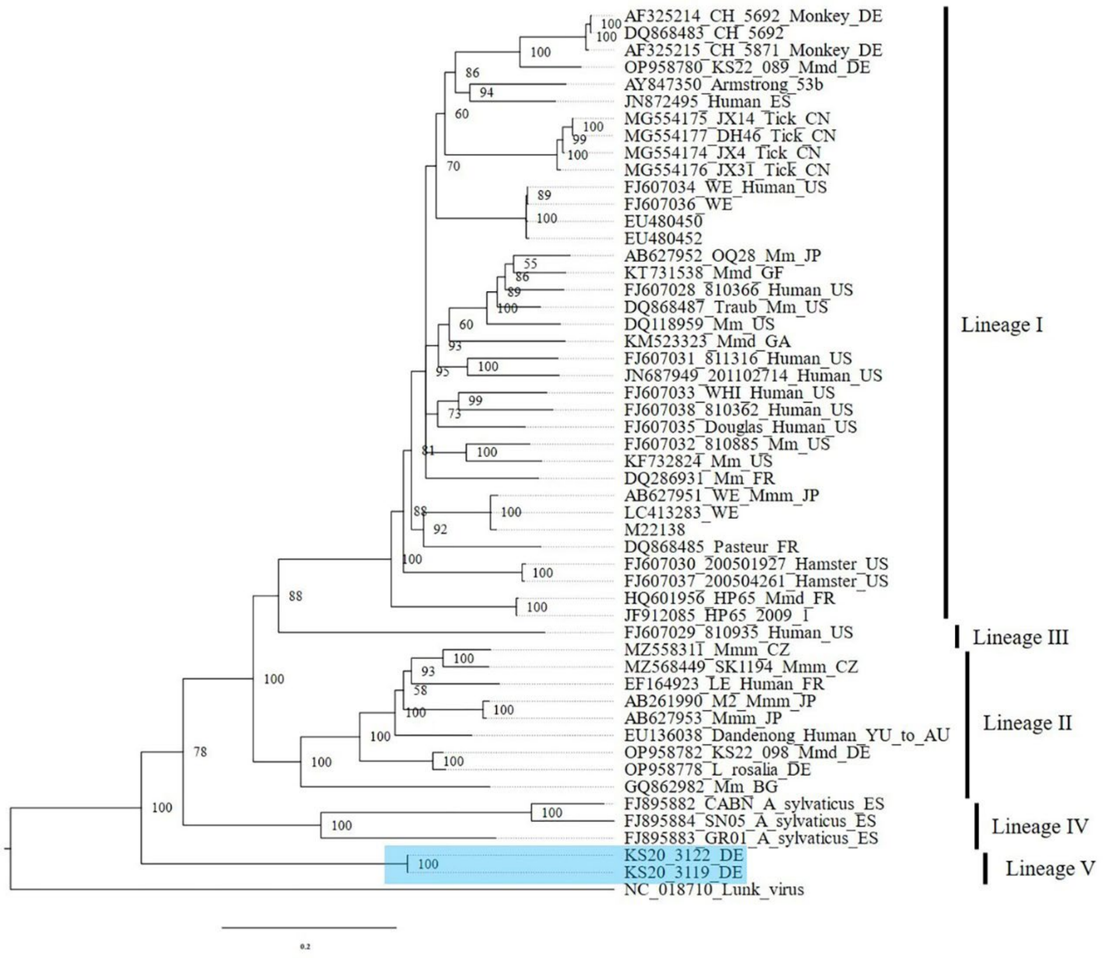

Appendix Figure 3. Phylogenetic analysis of the glycoprotein encoding region of lymphocytic choriomeningitis virus lineage V in wood mice, Germany. Bayesian inference method was used to analyze 1,494 nt corresponding to codons 1–498, excluding the stop codon. Sequences from this study are highlighted in blue. Lunk virus from *Mus minutoides* mice was used as an outgroup. Sequence names are comprised of the GenBank accession number, strain name, host species and country of origin (if known). Countries are represented by their international organization of standardization code. Roman numerals I–IV represent the different virus lineages as defined previously (*9*). WE and Armstrong are laboratory strains of lymphocytic choriomeningitis virus. Asyl, *Apodemus sylvaticus*; AU, Australia; BG, Bulgaria; CN, China; CZ, Czech Republic; DE, Germany; ES, Spain; FR, France; GA, Gabon; GF, French Guiana; JP, Japan; Mm, *Mus musculus*; Mmm, *Mus musculus musculus*; Mmd, *Mus musculus domesticus*; SK, Slovakia; US, United States; YU, former Yugoslavia. Scale bar indicates nucleotide substitutions per site.

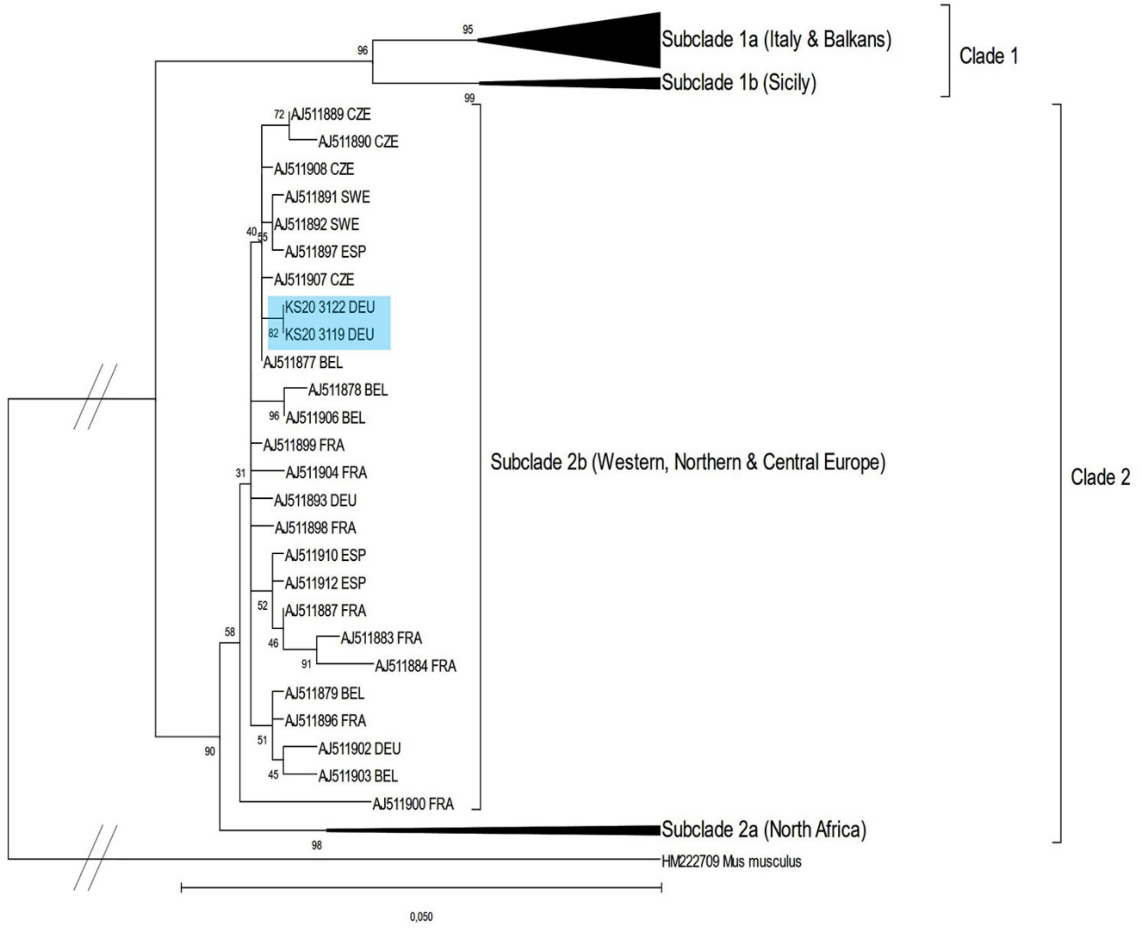

Appendix Figure 4. Phylogenetic analysis of mitochondrial cytochrome b DNA from *Apodemus sylvaticus* mice. Maximum-likelihood method was used for partial cytochrome b nucleotide sequences (909 nt, corresponding to codons 4718–5020). Sequences from this study are highlighted in blue. Sequence from *Mus musculus* mice was used as an outgroup. Sequence names are comprised of the GenBank accession number and country of origin (if known). Countries are represented by their international organization of standardization code. Clades are defined as previously described (*10*). Wood mouse phylogeny can be divided into 2 major clades according to mitochondrial cytochrome b DNA phylogenetics: clade 1 comprises populations from Italy, Sicily, and the Balkans and clade 2 comprises populations from North Africa and western, northern, and central Europe (*10*). Those clades can be further subdivided into subclades: subclade 1a, Italy and Balkans; 1b, Sicily; 2a, North Africa, and 2b, central Europe (*10*). Sequences obtained from wood mice from this study clustered in subclade 2b. Scale bar indicates nucleotide substitutions per site.
